# A cross-cultural validation of the Clinician Administered PTSD Scale for Children and Adolescents in a Dutch population

**DOI:** 10.3402/ejpt.v4i0.19896

**Published:** 2013-05-02

**Authors:** Julia Diehle, Carlijn de Roos, Frits Boer, Ramón J. L. Lindauer

**Affiliations:** 1 Department of Child and Adolescent Psychiatry, Academic Medical Center, Amsterdam, The Netherlands; 2 Psychotrauma Center for Children and Adolescents, MHI Rivierduinen, Leiden, The Netherlands; 3 De Bascule, Academic Center for Child and Adolescent Psychiatry, Amsterdam, The Netherlands

**Keywords:** Posttraumatic stress disorder, children, CAPS-CA, diagnostic interview, validity, reliability

## Abstract

**Background:**

Trauma-focused interventions for children could be administered more efficiently and effectively if posttraumatic stress disorder (PTSD) and related symptoms were first investigated by a reliable and valid instrument. The Clinician Administered PTSD Scale for Children and Adolescents (CAPS-CA) is the gold standard for the assessment of PTSD. Until now no cross-cultural validation study has been published in an English peer-reviewed journal.

**Objective:**

This study aimed at the cross-cultural validation of the Dutch CAPS-CA.

**Method:**

A total of 112 children between the age of 8 and 18 were recruited at two trauma centers. Children were interviewed with the CAPS-CA and the Anxiety Disorders Interview Schedule Child (ADIS-C) version, and each filled out the Children's Revised Impact of Events Scale (CRIES-13), the Revised Child Anxiety and Depression Scale (RCADS), and the Strength and Difficulties Questionnaire (SDQ). One caretaker of each child was also interviewed by means of the ADIS Parent (ADIS-P) version and filled out the RCADS and SDQ.

**Results:**

The Dutch CAPS-CA showed as good internal consistency, inter-rater reliability, convergent and divergent validity, and concurrent validity as the original English version. Similar to the original version, we found better psychometric properties in terms of internal consistency and convergent validity for children 13 years and older than for children younger than 13 years.

**Conclusions:**

The Dutch CAPS-CA is as reliable and valid as the original English version.

Children suffer from traumatic events like assault, sexual abuse, natural disasters, or accidents daily. Approximately 10–35% of these children develop a posttraumatic stress disorder (PTSD; Berman, Kurtines, Silverman, & Serafini, 1996; Kilpatrick et al., 2003).

Trauma-focused interventions can help children to reduce PTSD symptoms and impede negative consequences (National Institute for Clinical Excellence [NICE], 2005). These interventions could be administered more efficiently and effectively if a reliable and valid instrument first investigated PTSD and related symptoms. Clinical interviews can help in identifying trauma-reminders and avoidance behaviors or strategies. On the basis of this information, therapists can more rapidly target specific trauma-reminders and avoidance behaviors during therapy (March, 1999).

According to the NICE guidelines (2005), the Clinician Administered PTSD Scale for Children and Adolescents (CAPS-CA; Nader et al., 2006) is the gold standard for assessing PTSD symptoms with a structured clinical interview. Nader et al. (2006) adapted the CAPS adult version (Blake et al., 1995) to fit the specific needs of children. Besides linguistic changes, the authors included child-specific symptoms and rating tools. Due to its ability to measure symptom severity and treatment outcome, the original English version of the CAPS-CA has proven useful in a variety of studies including randomized controlled trials and neurobiological and prediction studies (e.g., Carrion, Weems, Richert, Hoffman, & Reiss, 2010; Daviss et al., 2000; Smith et al., 2007;). The continuous outcome for symptom severity creates extra possibilities for analyzing and interpreting outcome data. Carrion, Weems, Ray, and Reiss (2002), Saltzman, Weems, and Carrion (2006), and Erwin, Newman, McMackin, Morrissey, and Kaloupek (2000) also investigated the psychometric properties of the CAPS-CA in their studies. They found that the interview showed good internal consistency, acceptable convergent validity with other measures of PTSD like the Childhood Posttraumatic Stress Reaction Index (CPTSD-RI) or the PTSD checklist, and good inter-rater reliability. Harrington (2009) found in her validation study that the CAPS-CA also had good divergent validity. This was displayed by lower correlations with measures of depression (Beck Depression Inventory II, BDI), anxiety (Revised Children's Manifest Anxiety Scale, RCMAS), and behavior and emotional problems (Youth Self Report, YSR) than with self-report measures of PTSD (Child PTSD Symptom Scale, CPSS, and Children's PTSD Inventory). Given these excellent properties, the CAPS-CA almost fulfills the wish list for an ideal pediatric PTSD assessment tool as described by March (1999). Until now however the CAPS-CA has proven its qualities particularly in English-speaking populations.

To our knowledge, the CAPS-CA has only been cross-culturally validated in Germany by Steil & Füchsel (2006) and in Turkey by Karakaya et al. (2007). Researchers have also used the CAPS-CA in German-speaking populations (Wittmann, Zehnder, Jenni, & Landolt, 2012). Unfortunately, the validation studies themselves have not yet been published in English peer-reviewed journals, and cross-cultural validation results are therefore not available to the majority of clinical and research public. With the present research, we aimed at filling this gap and by doing so also aimed at presenting a valid instrument for the assessment of PTSD for the Dutch child population. Hence, the goal of our research was to cross-culturally validate the Dutch version of the CAPS-CA. For this purpose, we examined: 1) the internal consistency of the CAPS-CA and its inter-rater reliability; 2) its convergent validity through its correlations with the Children's Revised Impact of Event Scale 13-items version (CRIES-13; Dyregrov & Yule, 1995) and its agreement with the Anxiety Disorder Interview Schedule for DSM-IV: Child and Parent interview schedule (ADIS-C/P; Silverman & Albano, 1996); 3) its divergent validity through its correlations with the Revised Child Anxiety and Depression Scale (RCADS; Chorpita, Yim, Moffitt, Umemoto, & Francis, 2000) and the Strengths and Difficulties Questionnaire (SDQ; Goodman, 1997); and 4) its concurrent validity by investigating the difference in CAPS-CA total score from pre- to post-treatment and investigating the difference between children who experienced an event which fulfills the A criterion as described in the Diagnostic Statistical Manual of Mental Disorders (DSM-IV-TR; American Psychiatric Association [APA], 2000) and children who experienced an event which did not fulfill the A criterion. We hypothesized that the Dutch version of the CAPS-CA has as good psychometric qualities as the original English version.

## Method

### Participants

Our final sample consisted of 112 children, with 105 treatment seekers at two centers for child and adolescent psychiatry (de Bascule; child and adolescent psychiatry of the Academic Medical Center (AMC) in Amsterdam, and the Mental Health Institution Rivierduinen; child and adolescent department in Leiden) and seven children who were screened for PTSD after having been treated at the emergency department of the AMC. We interviewed 34 of the treatment-seeking children for a second time post-treatment. A total of 102 caregivers were willing to participate in the interview and/or to fill out the questionnaires.

Children were excluded from the study for the following reasons: being younger than 8 or older than 18 years; had sought treatment less than a month after the adverse event; were diagnosed with a present or past diagnosis of schizophrenia; and were not able to complete the CAPS-CA interview due to insufficient knowledge of the Dutch language.

The mean age was 12.92 years (SD=3.44, range 8–18 years). Further demographics are presented in Table 1. Children were exposed to a variety of adverse events. Most frequent single traumatic events were: traffic accident (15.2%), sexual abuse (8%), and assault with a weapon (7%). Sexual abuse and domestic violence were the most frequently reported chronic traumatic events, with 11.6% and 8.3%, respectively.

**Table 1:** Demographics

Variable	Frequency	%
Sex		
Male	48	42.9
Female	64	57.1
Ethnicity child		
Dutch	91	81.3
European (other)	5	4.5
Moroccan	3	2.7
African (other)	3	2.7
Latin American	2	1.8
Asian	2	1.8
North American	1	0.9
Missing	5	4.5
Ethnicity mother		
Dutch	63	56.3
Moroccan	11	9.8
Suriname	9	8.0
European (other)	9	8.0
African (other)	6	5.4
Latin American	2	1.8
Asian	3	2.7
Missing	9	8.0
Ethnicity father		
Dutch	56	50.0
Moroccan	13	11.6
Suriname	11	9.8
European (other)	8	7.1
African (other)	6	5.4
Latin American	2	1.8
Asian	2	1.8
Dutch Antillean	1	0.9
Missing	13	11.6
Core adverse event		
Non-traumatic	15	13.4
Single traumatic event	57	50.9
Multiple traumatic events	40	35.7
Living situation		
Single parent household	38	33.9
Two parents household	38	33.9
Foster home	17	15.2
Other	8	7.1
Crisis center	2	1.8
Missing	9	8.0

## Procedure

Children and their caretakers were asked for their participation during the standard intake procedure. Those willing to participate signed an informed consent form. At T1, trained psychologists administered the CAPS-CA. Approximately 3–10 days later (T2), a psychologist who was unaware of the result of the CAPS-CA, administered the ADIS-C to the child. One caretaker was interviewed by means of the ADIS-P at either T1 or T2. Questionnaires were administered to the child and the interviewed caretaker at T1 or T2.

Data were collected as part of a larger clinical study. This study has been approved by the local ethical committee.

## Measures

### CAPS-CA (Nader et al., 2006)

The CAPS-CA is a standardized clinical interview developed to assess PTSD conform the DSM-IV-TR standards. The duration of the interview can vary between 30 and 75 minutes, depending on the age and trauma history of the child.

The core traumatic event is chosen on the basis of the life-events checklist, which inquires about various possible traumatic events. If the core traumatic event is actually a sequence of events, which is often the case in domestic violence, a brief term is chosen to capture the core of these events.

For each of the 17 PTSD symptoms, the interviewer can rate the frequency and the intensity on a five-point Likert scale. This allows the interviewer to give a more sophisticated answer as to how often (from 0 “none of the time” to 4 “most of the time, daily or almost every day”) the patient was troubled by a symptom and to what extent the patient considered this symptom as problematic (0 “not a problem” to 4 “a whole lot, extreme, incapacitating distress, had to stop activity”). The sum of the frequency and intensity score gives the severity score for each item. The overall severity score for all 17 symptoms can vary between minimal (<20) and extreme (>79–136). Besides this continuous score, the chosen scoring rule also allows for a binary score (present or absent) of each symptom. In our study, we adopted the most frequently used scoring rule “frequency at least 1 and intensity at least 2” as proposed by Weathers, Ruscio, and Keane (1999) to score a symptom as being present.

For the present study, the original English version of the CAPS-CA was translated into Dutch by a group of native Dutch-speaking child psychologists and psychiatrists. The Dutch version was then back-translated into English by a native English speaker who is also a professional translator. The back-translated version was sent to the original authors of the CAPS-CA who approved the version after some minor changes.

The original English version of the CAPS-CA has shown poor to good internal consistency with coefficient αs varying between 0.52 and 0.82 in child populations in the age range of 7–14 (Saltzman et al., 2006) and acceptable to excellent coefficient αs varying between 0.72 and 0.9 in child populations older than 14 (Erwin et al., 2000; Harrington, 2009). Inter-rater reliability was excellent (intraclass correlation coefficient [ICC]=0.97; Carrion et al., 2002). Carrion et al. (2002) demonstrated for children in the age range of 7–14 years that the CAPS-CA has acceptable convergent validity by correlating significantly with the Reaction Index (r=0.51). In child populations older than 14, Erwin et al. (2000) and Harrington (2009) found that the CAPS-CA correlated significantly with the PTSD checklist (r=0.64) and with the Child PTSD Inventory (0.74).

Mean scores on the CAPS-CA vary between studies. Carrion, Haas, Garrett, Song, & Reiss (2010) ,for example, found a mean CAPS-CA score of 44.6 in a sample of children with a history of interpersonal trauma and who suffered from partial or full PTSD. Daviss et al. (2000) found in a sample of injured pediatric patients without PTSD, partial PTSD and full PTSD an overall mean score of 24.4. In our study we also included children without PTSD, partial and full PTSD and found a mean score of 32.99.

### ADIS-C/P (Silverman & Albano, 1996)

The ADIS-C/P is a structured clinical interview that can be used to assess anxiety and mood disorders in children and adolescents. The different versions can be administered to the child him or herself or to the caretaker. Symptoms can be rated as either present or absent. Several modules of the original English version have been validated. Unfortunately psychometric properties about the PTSD module of the ADIS-C/P have to our knowledge not yet been investigated. However, since the ADIS-C/P is a widely used clinical interview, we administered the PTSD module to children and one caretaker, independently of each other in the current study.

### CRIES-13 (Dyregrov & Yule, 1995)

The CRIES-13 is a screenings tool for PTSD symptoms. The questionnaire consists of 13 items, which are clustered in three subscales: avoidance, re-experiencing and arousal. Items can be rated as not at all (0), rarely (1), sometimes (3) and often (5). The CRIES-13 has shown good internal consistency for the total scale: coefficient α=0.8 and satisfactory internal consistency for the three subscales: intrusions coefficient α=0.7, avoidance coefficient α=0.73, and arousal coefficient α=0.6 (e.g., Smith, Perrin, Dyregrov, and Yule, 2003). In our sample, we found coefficient α=0.87 for the total scale and 0.79, 0.73 and 0.7 for the three subscales intrusion, avoidance, and arousal, respectively.

### RCADS (Chorpita et al., 2000)

The RCADS is a 47-item questionnaire that inquires about symptoms of anxiety and depression. In the current study, we used both the child and the parent version.

Chorpita et al. (2000) found that the subscales of the child questionnaire had good internal consistencies, with coefficient αs ranging between 0.71 and 0.85. Ebesutani, Bernstein, Nakamura, Chorpita, and Weisz (2010) validated the English parent version of the RCADS and also found good internal consistencies for the six subscales with coefficient αs ranging between 0.81 and 0.94. In our study, the internal consistencies of the subscales for the child version were: 0.87 for social phobia (SP); 0.88 for panic disorder (PD); 0.83 for generalized anxiety disorder (GAD); 0.91 for major depressive disorder (MDD); 0.75 for separation anxiety disorder (SAD); and 0.75 for obsessive compulsive disorder (OCD). Cronbach's αs for the parent version were 0.89 for SP; 0.86 for PD; 0.85 for GAD; 0.86 for MDD; 0.81 for SAD; and 0.75 for OCD.

### SDQ (Goodman, 1997)

The SDQ is a brief behavioral screening questionnaire with five subscales. The questionnaire is validated for children between 11 and 16 years. The Dutch version of the SDQ was validated by Widenfelt, Goedhart, Treffers, and Goodman (2003). They found good internal consistencies for both the child and the parent version. In their study, Cronbach's αs of the subscales ranged between 0.57 and 0.84 for the child version; and between 0.39 and 0.66 for the five subscales of the parent version. In our sample, Cronbach's αs for the five subscales of the child version were: 0.75 for emotional problem (EP); 0.37 for conduct problems (CP); 0.69 for hyperactivity/inattention (HI); 0.4 for peer problems (PP); and 0.41 for prosocial scale (PS). For the parent version, we found EP: 0.77; CP: 0.49; HI: 0.77; PP: 0.6; and PS: 0.6.

## Data analysis

All data were analyzed with SPSS version 19. Two children could/would not answer one of the questions of the CAPS-CA. Therefore, these two cases are excluded from the total scale analysis. A total of 97 children filled out the CRIES-13. A total of 36 participants did not return the RCADS questionnaire, which left us with *n*=93 for the child and *n*=85 for the parent version. The SDQ was filled in by 69 children (only children older than 11 filled in the child version) and 87 caretakers. For the correlation analyses, we imputed missing items on the subscale level if less than 20% was missing. For the ADIS-C, *n*=105, and for the ADIS-P, *n*=99.

### Reliability analysis

For the investigation of the reliability of the CAPS-CA, we calculated Cronbach's αs for the total scale and the three subscales, using the severity score for each item. Given that previous studies found higher Cronbach's αs in older children than in younger children (Erwin et al., 2000; Harrington, 2009; Saltzman et al., 2006) we performed separate analyses for children in the age ranges 8–12 and 13–18. From George and Mallery (2003), we interpreted coefficient αs <0.5 as unacceptable, ≥0.5 but <0.6 as poor, ≥0.6 but<0.7 as questionable, ≥0.7 and<0.8 as acceptable, ≥0.8 but<0.9 as good, and ≥0.9 as excellent.

We calculated Fleiss’ generalized κ for the inter-rater agreement between four interviewers on a random selection of 24 interviews (21% of the total amount of the conducted interviews). Following the suggestions of Fleiss (1981), we interpreted κ coefficients<0.4 as poor, coefficients ≥0.4 but ≤0.75 as fair to good, and>0.75 as excellent. Furthermore, we calculated the ICC between the four interviewers on the total CAPS-CA score of the same 24 interviews. The ICC was interpreted in the same way as the κ coefficient.

### Validity analysis

The convergent validity was investigated by correlating the CAPS-CA with the CRIES-13. Furthermore, we calculated the agreement between the CAPS-CA diagnosis and the ADIS-C diagnosis and the ADIS-P diagnosis by means of the κ coefficient. Results were interpreted as described earlier. We performed separate analyses for children aged 8–12 and 13–18, since past studies have demonstrated that the CAPS-CA showed higher correlations with related measures in older children than in younger children (Carrion et al., 2002; Erwin et al., 2000; Harrington, 2009).

For the divergent validity, we calculated the correlations of the CAPS-CA with the RCADS subscale scores; and the SDQ subscale scores. We used Pearson product moment correlations for all correlation analyses. According to Cohen (1988) correlations,<0.30 are considered small, correlations ≥0.30 and<0.50 are considered medium, and ≥0.50 are considered strong.

We investigated the concurrent validity of the CAPS-CA by comparing different groups to each other: we conducted the paired sample *t*-test for pre-treatment to post-treatment changes; and we used a one sample *t*-test for the comparison of children who were exposed to an event which did not fulfill the A criterion according to DSM-IV-TR standards and children who were exposed to an event which did fulfill the A criterion.

## Results

### Reliability analysis

Table 2 presents Cronbach's αs and item total correlations for the three subscales and the total scale of the CAPS-CA for the whole sample and clustered by age group. We found the lowest coefficient α of 0.42 for cluster C in the age group 8–12 and the highest coefficient α of 0.86 for the total scale in the age group 13–18. We found excellent inter-rater reliability with an ICC for the total scale of 0.99. For the frequency and intensity subscales, we found ICCs of 0.99 and 0.97, respectively. Fleiss’ generalized κ was 0.75 for the agreement on PTSD diagnosis between the four interviewers.

**Table 2:** Item total correlations and Cronbach's αs for CAPS-CA total and subscales for the total sample and per age group

CAPS-CA	Sample	Item to total correlations	Cronbach's α
Cluster B	Total	0.31–0.66	0.75
	8–12	0.09–0.62	0.72
	13–18	0.34–0.69	0.78
Cluster C	Total	0.15–0.56	0.64
	8–12	−0.03–0.40	0.42
	13–18	0.13–0.57	0.68
Cluster D	Total	0.28–0.57	0.62
	8–12	0.24–0.44	0.55
	13–18	0.29–0.65	0.67
Total scale	Total	0.15–0.67	0.83
	8–12	0.02–0.66	0.77
	13–18	0.14–0.73	0.86

### Validity analysis

The CAPS-CA showed medium to strong correlations with the CRIES-13 (see Table 3). We found the smallest correlation for cluster C in the age group 8–12. The strongest correlation was for the total scores in the age group 13–18.

**Table 3:** Pearson correlation coefficients between CAPS-CA and CRIES-13

Sample	Cluster B	Cluster C	Cluster D	Total score
Total	0.66**	0.38**	0.58**	0.67**
8–12	0.61**	0.35*	0.57**	0.57**
13–18	0.71**	0.48**	0.59**	0.73**

* Correlation is significant at the 0.05 level (2-tailed).

** Correlation is significant at the 0.01 level (2-tailed).

The coefficient κs for the agreement in cluster fulfillment and PTSD diagnosis of the CAPS-CA and the ADIS-C and P varied between fair and poor with the lowest agreements on cluster C (see Table 4) and the highest agreements on cluster B.

**Table 4:** Kappa coefficients between CAPS-CA and ADIS-C/P

	Sample	κ CAPS-CA and ADIS-C	κ CAPS-CA and ADIS-P	κ ADIS-C and ADIS-P
Cluster B	Total	0.47**	0.44**	0.32**
	8–12	0.33*	0.65**	0.23
	13–18	0.59**	0.27	0.45**
Cluster C	Total	0.27**	0.19*	0.07
	8–12	0.17	0.18	−0.06
	13–18	0.31**	0.19	0.19
Cluster D	Total	0.42**	0.20*	0.23*
	8–12	0.43**	0.19	0.27*
	13–18	0.41**	0.22	0.14
Diagnosis	Total	0.32**	0.21*	0.12
	8–12	0.16	0.29*	0.00
	13–18	0.34**	0.14	0.23

* κ coefficient is significant at the 0.05 level (2-tailed).

** κ coefficient is significant at the 0.01 level (2-tailed).

Tables 5 and 6 show the correlations of the CAPS-CA total scale and subscales with the RCADS subscales and the SDQ subscales. Correlations with the RCADS and SDQ subscales were moderate to strong with some exceptions: the CAPS-CA showed small correlations with the RCADS parent subscale “social phobia” and the SDQ subscales, “conduct problems” and “prosocial scale” for both the child and parent versions. The highest correlation between the CAPS-CA and the RCADS or SDQ was between the CAPS-CA total score and the RCADS child version “depression subscale” (r=0.59).

**Table 5:** Correlation coefficients between CAPS-CA and other child measures

	Mean	SD	CAPS-CA total	CASPS-CA intrusions	CAPS-CA avoidance	CAPS-CA hyperarousal
CAPS-CA total	32.99	20.51	1	0.85**	0.83**	0.82**
CAPS-CA intrusions	11.37	8.22	0.85**	1	0.56**	0.55**
CAPS-CA avoidance	10.32	8.22	0.83**	0.56**	1	0.5**
CAPS-CA hyperarousal	11.27	8.02	0.82**	0.55**	0.5**	1
RCADS separation anxiety	4.08	3.65	0.4**	0.48**	0.3**	0.2
RCADS social phobia	8.65	5.68	0.35**	0.32**	0.41**	0.13
RCADS generalized anxiety	4.98	3.61	0.53**	0.48**	0.47**	0.35**
RCADS panic disorder	4.94	4.77	0.56**	0.55**	0.48**	0.35**
RCADS OCD	4.01	3.44	0.49**	0.51**	0.42**	0.28**
RCADS depression	8.33	6.23	0.59**	0.53**	0.49**	0.43**
SDQ emotional problems	4.10	2.72	0.57**	0.53**	0.58**	0.36**
SDQ conduct problems	2.04	1.38	0.10	0.13	−0.01	0.13
SDQ hyperactivity	4.32	2.21	0.47**	0.38**	0.42**	0.40**
SDQ peer problems	2.57	1.84	0.23	0.11	0.35**	0.13
SDQ prosocial behavior	8.29	1.34	0.02	0.03	0.03	−0.02
SDQ total difficulties score	13.05	5.34	0.58**	0.49**	0.58**	0.43**

** Correlation is significant at the 0.01 level (2-tailed).

**Table 6:** Correlation coefficients between CAPS-CA and parent measures

	Mean	SD	CAPS-CA total score	CAPS-CA intrusions	CAPS-CA avoidance	CAPS-CA hyperarousal
RCADS separation anxiety	4.56	4.01	0.34**	0.35**	0.27*	0.21
RCADS social phobia	8.72	5.57	0.21	0.18	0.26*	0.10
RCADS generalized anxiety	4.82	3.51	0.46**	0.39**	0.37**	0.38**
RCADS panic disorder	3.51	3.97	0.42**	0.41**	0.39**	0.24*
RCADS OCD	2.25	2.68	0.42**	0.38**	0.36**	0.31**
RCADS depression	7.58	5.29	0.46**	0.37**	0.47**	0.30**
SDQ emotional problems	3.87	2.71	0.33**	0.23*	0.36**	0.25*
SDQ conduct problems	2.12	1.69	0.04	0.05	0.03	0.03
SDQ hyperactivity	4.65	2.67	0.10	0.05	0.11	0.09
SDQ peer problems	2.29	2.06	0.22*	0.13	0.24*	0.18
SDQ prosocial behavior	8.28	1.70	−0.10	−0.10	−0.13	−0.02
SDQ total difficulties score	12.92	6.12	0.28**	0.18	0.30**	0.22*

* Correlation is significant at the 0.05 level (2-tailed).

** Correlation is significant at the 0.01 level (2-tailed).

The paired samples *t*-test of pre-and post-test scores of children who received trauma therapy was significant: *t*(33)=6.56, *p*=0.00. Children's pre-treatment mean score on the CAPS-CA was M=41.12 (SD=17.72) and for post-treatment was M=18.59 (SD=22.24).

Furthermore we compared those children who experienced an event which fulfilled the A criterion of PTSD according to the DSM-IV-TR standards (*n*=98) to those children whose event did not fulfill the A criterion (*n*=14). The independent samples *t*-test revealed significantly higher scores for the children who did fulfill the A criterion, M=35.15 (SD=20.37), *t*(110)=−3.06, *p*=0.00 than for children whose experience did not fulfill the A criterion, M=17.86 (SD=14.69).

## Discussion

Our cross-cultural validation study suggests that the psychometric properties of the Dutch version of the CAPS-CA are as good as those of the English version.

For the reliability analyses, we found Cronbach's αs, which can be interpreted as questionable to good, for the total sample. Differentiation for age groups revealed that coefficient αs for the total scale were comparable to coefficient αs found by Saltzman et al. (2006) for younger children and comparable to Harrington (2009) for older children. The two age groups in our sample differed especially in the Cronbach's αs for the avoidance and numbing subscale. Cronbach's α for this cluster was lower for younger children than for older children. Item C2 (“Efforts to avoid activities, places, or people that arouse recollections of the trauma”) seems particularly responsible for the difference. Cronbach's α of the subscale would rise up to 0.5 after deletion of this item. A possible explanation for this could be that younger children in our sample were less able to avoid activities or places that remind them of the traumatic event than older children were. Another reason for the low coefficient α of this symptom cluster could be that younger children had more difficulties to understand these questions than older children. Further research in younger children is needed to investigate the construct of cluster C itself and the understanding of these items.

Our results from the inter-rater analyses confirm that the CAPS-CA has good inter-rater reliability. The ICC for 4 interviewers was excellent for the total as well as for the frequency and intensity subscale scores (0.99, 0.99, and 0.97, respectively). Given that, the Dutch version is also comparable with the English version (Carrion et al., 2002). The κ coefficient of 0.75 for the agreement on a diagnosis on the CAPS-CA between the four raters was also good.

Taken together, our findings on the reliability of the Dutch CAPS-CA suggest that it is more reliable for older children than for younger children. These findings are consistent with previous findings in English-speaking child and adolescent populations.

Considering the convergent validity of the CAPS-CA, our correlations of the CAPS-CA subscales and total scale and the corresponding scales on the CRIES-13 can be interpreted as strong. For the younger age group, we found a correlation for the total scale that was higher than the one found by Carrion et al. (2002) with the Reaction Index. Our correlation of the total scale for the older age group is higher than the correlation found by Erwin et al. (2000) with the PTSD checklist and is comparable to the correlations found by Harrington (2009) with the Child PTSD Inventory. These findings indicate that the Dutch CAPS-CA has slightly better convergent validity than the original English version.

We found fair to poor agreement between the CAPS-CA and ADIS-C/P considering PTSD diagnoses and cluster fulfillment. We had expected somewhat better agreement between the two instruments given that they both argue to investigate PTSD according to DSM-IV-TR standards (APA, 2000). We think that there are two reasons that may explain this discrepancy: First, the ADIS-C/P is limited in the scoring of a symptom as present or absent whereas the CAPS-CA also investigates the frequency and intensity of each symptom. A symptom that causes only mild problems might be scored present with the ADIS but not with the CAPS-CA since we chose the scoring rule in which a symptom had to cause at least moderate problems to be scored as present. The second reason lies in the construction of cluster C of the ADIS-C/P interview. Questions concerning item C4, diminished interest in activities, and C6, restricted range in affect, are phrased quite differently in the ADIS and the CAPS-CA. Furthermore, with the CAPS-CA the interviewer adheres strictly to the seven symptoms as presented in the DSM-IV-TR (APA, 2000), while in the ADIS-C/P interview, impairment in developmental functioning is also scored as a symptom within cluster C. Following the DSM-IV-TR criteria, this item should be scored under criterion F as is the case in the CAPS-CA. These differences may contribute to the low agreement between the ADIS and the CAPS-CA, especially on cluster C.

The correlations between the CAPS-CA and the RCADS and SDQ subscales suggest that the divergent validity of the CAPS-CA is also satisfying. Although most of the correlations were also significant, none was as high as the correlation between the CAPS-CA total scale and the CRIES-13 total scale. Like Harrington (2009), we found relatively high, significant correlations between the CAPS-CA and self-report measures of depression. Both the child and the parent subscale for depression on the RCADS correlated strongly with the CAPS-CA. These strong correlations between the CAPS-CA and the depression subscales are yet another indication for the validity of the CAPS-CA when bearing in mind that PTSD and major depressive disorder have great overlap in symptomatology according to the DSM-IV-TR standards (APA, 2000). Significant correlations between the CAPS-CA and measures of other anxiety disorders or hyperactivity can also be explained by symptom-overlap but also by the high co-morbidity of PTSD and other anxiety disorders.

For the concurrent validity, we found that the CAPS-CA is able to measure treatment effects. Children had significantly lower scores on the CAPS-CA after trauma therapy than before therapy. Furthermore, we found that the CAPS-CA is able to distinguish between those children whose experience did not meet the A criterion and those whose experience met the A criterion. Children whose experience cannot be classified as traumatic according to DSM-IV-TR standards (APA, 2000) scored significantly lower than children whose experience was classified as traumatic. This again reflects the good correspondence of the CAPS-CA with the current DSM-IV-TR criteria.

## Limitations

Our study suffered from several limitations: We collected our data at two trauma centers within a limited period of time. This resulted in a small, convenience sample that limits the generalizability of our results.

Another limitation involves our use of the CRIES-13 and ADIS-C/P for investigating the convergent validity of the CAPS-CA. Both measures are limited in their investigation of PTSD symptoms and are both not validated in the Dutch language. However, they are widely used and validated instruments for the investigation of PTSD in children are scarce in the Dutch language. Furthermore, we were not able to investigate the test–retest reliability of the CAPS-CA in our study. Given that the great majority of our sample consisted of treatment-seeking children and adolescents, we did not consider it appropriate to interview them twice with the CAPS-CA before treatment.

## Clinical implications and future directions

The good correspondence with the DSM-IV criteria and the continuous outcome make the CAPS-CA an attractive instrument when the primary interest of the interviewer lies in the exploration of PTSD and the measurement of treatment outcome. However, when more information about co-morbid disorders is required, questionnaires or interviews like the ADIS-C/P can be helpful. This is also the case when information from caretakers is needed. Unlike the ADIS, there is no version of the CAPS-CA that can be administered to caretakers. Such a version might be very helpful to get structured information from another source, especially when children are younger than 12.

Like many other instruments, the CAPS-CA will need adaptation when the new DSM 5 criteria will be published. Since one of the biggest advantages of the CAPS-CA over other instruments is its adherence to current diagnostic criteria, the interview should be adapted to reflect the DSM 5 criteria as soon as possible.

## Conclusion

The current study led to the first validated semi-structured clinical interview for the investigation of PTSD in the Dutch child and adolescent population. We can conclude that the Dutch version of the CAPS-CA has as good psychometric properties as the original English version. Future studies should investigate the properties of the CAPS-CA in other populations and should also investigate the test–retest reliability of the CAPS-CA.
